# Regionally decreased cortical gyrification in patients with major depressive disorder

**DOI:** 10.1192/j.eurpsy.2023.892

**Published:** 2023-07-19

**Authors:** K.-M. Han, Y. Kang, B.-J. Ham

**Affiliations:** 1 Department of Psychiatry, Korea University Anam Hospital, Korea University College of Medicine; 2 Department of Biomedical Sciences, Korea University College of Medicine, Seoul, Korea, Republic Of

## Abstract

**Introduction:**

Early neurodevelopmental deviations, such as abnormal cortical folding patterns, are a candidate biomarker for major depressive disorder (MDD). Previous studies on patterns of abnormal cortical gyrification in MDD have provided valuable insights; however, the findings on cortical folding are controversial.

**Objectives:**

We aimed to investigate the association of MDD with the local gyrification index (LGI) in each cortical region at the whole-brain level and the association of the LGI with clinical characteristics of MDD, including recurrence, remission status, illness duration, severity of depression, and medication status of patients with MDD.

**Methods:**

We obtained T1-weighted images of 234 patients with MDD and 215 healthy controls (HCs). LGI values were automatically calculated using the FreeSurfer software according to the Desikan–Killiany atlas. LGI values from 66 cortical regions in the bilateral hemispheres were analyzed. We compared the LGI values between the MDD and HC groups using the analysis of covariance, including patients’ age, sex, and years of education as covariates. The association between clinical characteristics and LGI values was investigated in the MDD group.

**Results:**

Compared with HCs, patients with MDD showed significantly decreased LGI values in the cortical regions, including the bilateral ventrolateral and dorsolateral prefrontal cortices, medial and lateral orbitofrontal cortices, insula, right rostral anterior cingulate cortex, and several temporal and parietal regions, with the highest effect size in the left pars triangularis (Cohen’s *f* = 0.361; P = 1.78 × 10^-13^). As for the association of clinical characteristics with LGIs within the MDD group, recurrence and longer illness duration of MDD were associated with increased gyrification in several occipital and temporal regions, which showed no significant difference in LGIs between MDD and HC groups.

**Conclusions:**

Considering that the aforementioned cortical regions are involved in emotion regulation, abnormal cortical folding patterns in such regions may be associated with the dysfunction of emotion regulation-related neural circuits, which may lead to MDD. These findings suggest that LGI may be a relatively stable neuroimaging marker associated with the trait of MDD predisposition.

**Disclosure of Interest:**

None Declared

